# Supplementary material for: Functional cross-talk between allosteric effects of activating and inhibiting ligands underlies PKM2 regulation
Source: eLife. 2019 Jul 2;8:e45068. doi: 10.7554/eLife.45068 (PMC6636998; doi:10.7554/eLife.45068)
Supplement: Supplementary file 2. [file elife-45068-supp2.docx]

**Human PKM2 as cloned in pET28a vector (His-tagged)**

**MGSSHHHHHHSSGLVPR||GSH**MSKPHSEAGTAFIQTQQLHAAMADTFLEHMCRLDIDSPPITARNTGIICTIGPASRSVETLKEMIKSGMNVARLNFSHGTHEYHAETIKNVRTATESFASDPILYRPVAVALDTKGPEIRTGLIKGSGTAEVELKKGATLKITLDNAYMEKCDENILWLDYKNICKVVEVGSKIYVDDGLISLQVKQKGADFLVTEVENGGSLGSKKGVNLPGAAVDLPAVSEKDIQDLKFGVEQDVDMVFASFIRKASDVHEVRKVLGEKGKNIKIISKIENHEGVRRFDEILEASDGIMVARGDLGIEIPAEKVFLAQKMMIGRCNRAGKPVICATQMLESMIKKPRPTRAEGSDVANAVLDGADCIMLSGETAKGDYPLEAVRMQHLIAREAEAAIYHLQLFEELRRLAPITSDPTEATAVGAVEASFKCCSGAIIVLTKSGRSAHQVARYRPRAPIIAVTRNPQTARQAHLYRGIFPVLCKDPVQEAWAEDVDLRVNFAMNVGKARGFFKKGDVVIVLTGWRPGSGFTNTMRVVPVP

In **RED**: His tag and linker from vector

In **BLACK**: human PKM2 (Uniprot entry: P14618)

Thrombin recognition site underlined and cleavage site denoted with **||**.

Theoretical average mass of thrombin-cleaved PKM2: 58218.16
