# Supplementary material for: Functional cross-talk between allosteric effects of activating and inhibiting ligands underlies PKM2 regulation
Source: eLife. 2019 Jul 2;8:e45068. doi: 10.7554/eLife.45068 (PMC6636998; doi:10.7554/eLife.45068)
Supplement: Transparent reporting form [file elife-45068-transrepform.docx]

***eLife’s* transparent reporting form**

We encourage authors to provide detailed information *within their submission* to facilitate the interpretation and replication of experiments. Authors can upload supporting documentation to indicate the use of appropriate reporting guidelines for health-related research (see [EQUATOR Network](http://www.equator-network.org/%20)), life science research (see the [BioSharing Information Resource](https://biosharing.org/)), or the [ARRIVE guidelines](http://www.plosbiology.org/article/info:doi/10.1371/journal.pbio.1000412) for reporting work involving animal research. Where applicable, authors should refer to any relevant reporting standards documents in this form.

If you have any questions, please consult our Journal Policies and/or contact us: [editorial@elifesciences.org](mailto:editorial@elifesciences.org).

**Sample-size estimation**

- You should state whether an appropriate sample size was computed when the study was being designed
- You should state the statistical method of sample size computation and any required assumptions
- If no explicit power analysis was used, you should describe how you decided what sample (replicate) size (number) to use

Please outline where this information can be found within the submission (e.g., sections or figure legends), or explain why this information doesn’t apply to your submission:

No explicit power analysis was used to estimate sample size because the number of replicates to test for statistical significance with the pre-defined significance and confidence criteria have been previously established, as reported in papers cited within the respective sections of the manuscript.

**Replicates**

- You should report how often each experiment was performed
- You should include a definition of biological versus technical replication
- The data obtained should be provided and sufficient information should be provided to indicate the number of independent biological and/or technical replicates
- If you encountered any outliers, you should describe how these were handled
- Criteria for exclusion/inclusion of data should be clearly stated
- High-throughput sequence data should be uploaded before submission, with a private link for reviewers provided (these are available from both GEO and ArrayExpress)

Please outline where this information can be found within the submission (e.g., sections or figure legends), or explain why this information doesn’t apply to your submission:

All information listed above can be found in the respective figure legends, table legends and Methods section.

**Statistical reporting**

- Statistical analysis methods should be described and justified
- Raw data should be presented in figures whenever informative to do so (typically when N per group is less than 10)
- For each experiment, you should identify the statistical tests used, exact values of N, definitions of center, methods of multiple test correction, and dispersion and precision measures (e.g., mean, median, SD, SEM, confidence intervals; and, for the major substantive results, a measure of effect size (e.g., Pearson's r, Cohen's d)
- Report exact p-values wherever possible alongside the summary statistics and 95% confidence intervals. These should be reported for all key questions and not only when the p-value is less than 0.05.

Please outline where this information can be found within the submission (e.g., sections or figure legends), or explain why this information doesn’t apply to your submission:

All information listed above can be found in the respective figure and table legends.

(For large datasets, or papers with a very large number of statistical tests, you may upload a single table file with tests, Ns, etc., with reference to sections in the manuscript.)

**Group allocation**

- Indicate how samples were allocated into experimental groups (in the case of clinical studies, please specify allocation to treatment method); if randomization was used, please also state if restricted randomization was applied
- Indicate if masking was used during group allocation, data collection and/or data analysis

Please outline where this information can be found within the submission (e.g., sections or figure legends), or explain why this information doesn’t apply to your submission:

The above information is not applicable to our study, which did not feature separately weighted experimental groups. Where different conditions were tested, equally-sized groups were used.

**Additional data files (“source data”)**

- We encourage you to upload relevant additional data files, such as numerical data that are represented as a graph in a figure, or as a summary table
- Where provided, these should be in the most useful format, and they can be uploaded as “Source data” files linked to a main figure or table
- Include model definition files including the full list of parameters used
- Include code used for data analysis (e.g., R, MatLab)
- Avoid stating that data files are “available upon request”

Please indicate the figures or tables for which source data files have been provided:

In relation to the computational results shown in Figure 4, we have deposited the software listed below at: [https://github.com/Fraternalilab/ALLOHUBMAT](https://emea01.safelinks.protection.outlook.com/?url=https%3A%2F%2Fgithub.com%2FFraternalilab%2FALLOHUBMAT&data=01%7C01%7Cfranca.fraternali%40kcl.ac.uk%7C6e285dd0d0804788cf2508d685d61956%7C8370cf1416f34c16b83c724071654356%7C0&sdata=tdy6gWLZ%2Ftpr5hLPE99N96%2BpL81zV578lTh5CitXyus%3D&reserved=0)

- sa_encode.R : Encode trajectory into stacked alignment of structural alphabet strings

- kabsch.R : Kabsch superpositioning routine

- MI.R : Mutual Information and other entropy metrics between two character vectors, here intended for two alignment columns
